# The Role of Diatom Nanostructures in Biasing Diffusion to Improve Uptake in a Patchy Nutrient Environment

**DOI:** 10.1371/journal.pone.0059548

**Published:** 2013-05-07

**Authors:** James G. Mitchell, Laurent Seuront, Mark J. Doubell, Dusan Losic, Nicolas H. Voelcker, Justin Seymour, Ratnesh Lal

**Affiliations:** 1 School of Biological Sciences, Flinders University, Adelaide, South Australia, Australia; 2 South Australian Research and Development Institute, Aquatic Sciences, West Beach, South Australia, Australia; 3 Centre National de la Recherche Scientifique, Laboratoire d'Océanologie et de Géosciences, Université des Sciences et Technologies de Lille, Station Marine, Wimereux, France; 4 School of Chemical Engineering, the University of Adelaide, Adelaide, South Australia, Australia; 5 Mawson Institute, University of South Australia, Adelaide, South Australia, Australia; 6 Plant Functional Biology & Climate Change Cluster, University of Technology, Sydney, New South Wales, Australia; 7 Department of Mechanical and Aerospace Engineering and Bioengineering, University of California San Diego, La Jolla, California, United States of America; University of Hull, United Kingdom

## Abstract

**Background:**

Diatoms are important single-celled autotrophs that dominate most lit aquatic environments and are distinguished by surficial frustules with intricate designs of unknown function.

**Principal Findings:**

We show that some frustule designs constrain diffusion to positively alter nutrient uptake. In nutrient gradients of 4 to 160 times over <5 cm, the screened-chambered morphology of *Coscincodiscus* sp. biases the nutrient diffusion towards the cell by at least 3.8 times the diffusion to the seawater. In contrast, the open-chambers of *Thalassiosira eccentrica* produce at least a 1.3 times diffusion advantage to the membrane over *Coscincodiscus* sp. when nutrients are homogeneous.

**Significance:**

Diffusion constraint explains the success of particular diatom species at given times and the overall success of diatoms. The results help answer the unresolved question of how adjacent microplankton compete. Furthermore, diffusion constraint by supramembrane nanostructures to alter molecular diffusion suggests that microbes compete via supramembrane topology, a competitive mechanism not considered by the standard smooth-surface equations used for nutrient uptake nor in microbial ecology and cell physiology.

## Introduction

Nutrient uptake is a crucial step for all cells. In microbial communities, multiple species compete for a single nutrient. For example, in the ocean, denitrifying bacteria, heterotrophic bacteria and phytoplankton can all vie for nitrate molecules. In this competitive environment eukaryotic phytoplankton are at a continuous disadvantage because bacteria are more than 90% of the biological surface area in the marine environment [1]. Diatoms in particular are at a disadvantage because they are not motile and so cannot vertically migrate, move to decaying fecal pellets and other distant source nutrient sources. Despite this disadvantage, diatoms are diverse and possibly the most widespread and successful of all phytoplankton [2]. The contradiction between the success of diatoms and their apparent disadvantages concerning uptake capability and immobility highlights a fundamental, unresolved question in aquatic microbial dynamics. How do diatoms compete with the surrounding, adjacent bacteria and motile phytoplankton for nutrients?

An obvious and early focus for studying competition for nutrients has been on the optimal and competitive tuning of high versus low affinity nutrient transporters [3]–[5]. While there is clearly affinity tuning, it does not explain all competitive interactions. If the cells with the fastest transporters, most transporters or greatest surface area to volume ratio were the most competitive strategies, bacteria would competitively exclude eukaryotic phytoplankton and diatoms in particular [3], [6]. How diatoms survive and seasonally flourish in competition with bacteria remains unresolved.

Diatoms, lacking motility, only have modifications of their surface as a means of competing. Their silica frustules permit shapes with large surface area to volume ratios compared to other more spherical groups such as bacteria and most eukaryotic phytoplankton. However, not all diatoms maximize their surface area to volume ratio by forming a thin disk. The one feature that is common to all diatoms is the surface structure, which is composed of micrometer and nanometer-sized shapes that create pores and chambers. This paper investigates whether these chambers may be the primary function of the frustule.

Is there any evidence that indicates frustule microstructure might be important for nutrient uptake and competition? There is indirect evidence in the form of the similarly disk shaped diatoms *Coscinodiscus* sp. and *Thalassiosira pseudonana* showing opposite growth responses to turbulence [7]. The difference in size did not explain all of the growth difference, and the authors could not find a methodological bias to explain why *Coscinodiscus* sp. grew faster in turbulence and *T. pseudonana* grew faster in a still environment [7]. Thus far, there is no explanation for linking their surface structure to their ecological success.

To explore the importance of surface structures we assess the extent to which the intricate structures on and in the diatom frustule contribute competitive advantage to the diatoms. We go on to estimate the extent of any advantage. To focus on microstructural differences and to address gaps pointed out in the literature [3]–[7], we compare the uptake dynamics of two diatoms with each other. This comparison also sheds light on how frustules might provide a competitive advantage in nutrient uptake to reduce or nullify the advantages bacteria have in receptor tuning, motility, numerical and surface area superiority.

The two criteria for determining whether frustules influence nutrient uptake are (1) the frustule structure must alter diffusion and (2) there must be fluctuations in nutrient concentrations at lengths relevant to individual cells. The evidence for molecular structure influencing diffusion is extensive, but has focused on bacteriophage and viral interactions with surfaces [8], [9] or molecular modification to diffusion within the cells [10]. Using principles and measurements from modeled nutrient gradients [11] and our measured nutrient gradients, we modeled the local environment above an individual diatom frustule to understand the relationship between nutrient change and nutrient uptake, with the goal of improving the understanding of the microenvironment where nutrient uptake occurs. We used modeling for the nutrient flux estimates because *in situ* uptake measurements through a single diatom frustule pore are well beyond current technology, as are *in situ* nutrient fluctuation measurements on the second time scale 1 µm above a diatom frustule.

For the criteria of fluctuations in nutrients, modeling predicts nutrient gradients are ubiquitous in the open, fully turbulent ocean down to 10 µm [11], but experimental evidence is lacking, even at millimeter and centimeter scales. In this context, we show that microscale inorganic nutrient gradients are a common feature of the Eastern English Channel. This tidally mixed coastal environment was chosen because it has continuously high dissipation rates that range from 5×10^−7^ to 5×10^−4^ m^2^ s^−3^ [12], which reflect the prevailing model for chemical distributions at small scales [11], and because *Coscinodiscus* sp. and *Thalasiosira* sp. co-exist in this region [13], [14].

Combining the two criteria, we demonstrate that, even in this well mixed environment, diatoms compete by the hitherto unappreciated mechanism of extracellular, transient trapping of nutrients. This transient trapping is micrometers above the diatom membrane within the frustule chambers, allowing diatoms to increase their own uptake and reduce the uptake of nearby competitors. This mechanism is distinct from, and superior to, nutrient adhesion to cell surfaces [15], because energetically expensive resolubilization is unnecessary. Our data suggest that extracellular transient trapping is a mechanistic explanation for why particular species and morphologies dominate at particular times. Finally, we point out the importance of sub-micrometer supramembrane structures in constraining diffusion, a principle that may apply in biology wherever nanometer to micrometer scale structures overlie membranes.

## Results

### Nutrients and phytoplankton gradients

**Figure 1:**
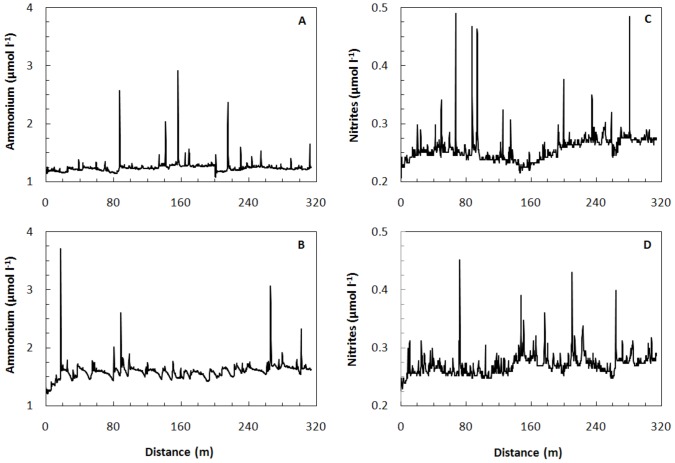
Ammonium and nitrite gradients in the Eastern English Channel at 3 s intervals. The steepest and largest gradients are at the shortest scales, those most relevant to individual phytoplankton cells.

**Figure 2:**
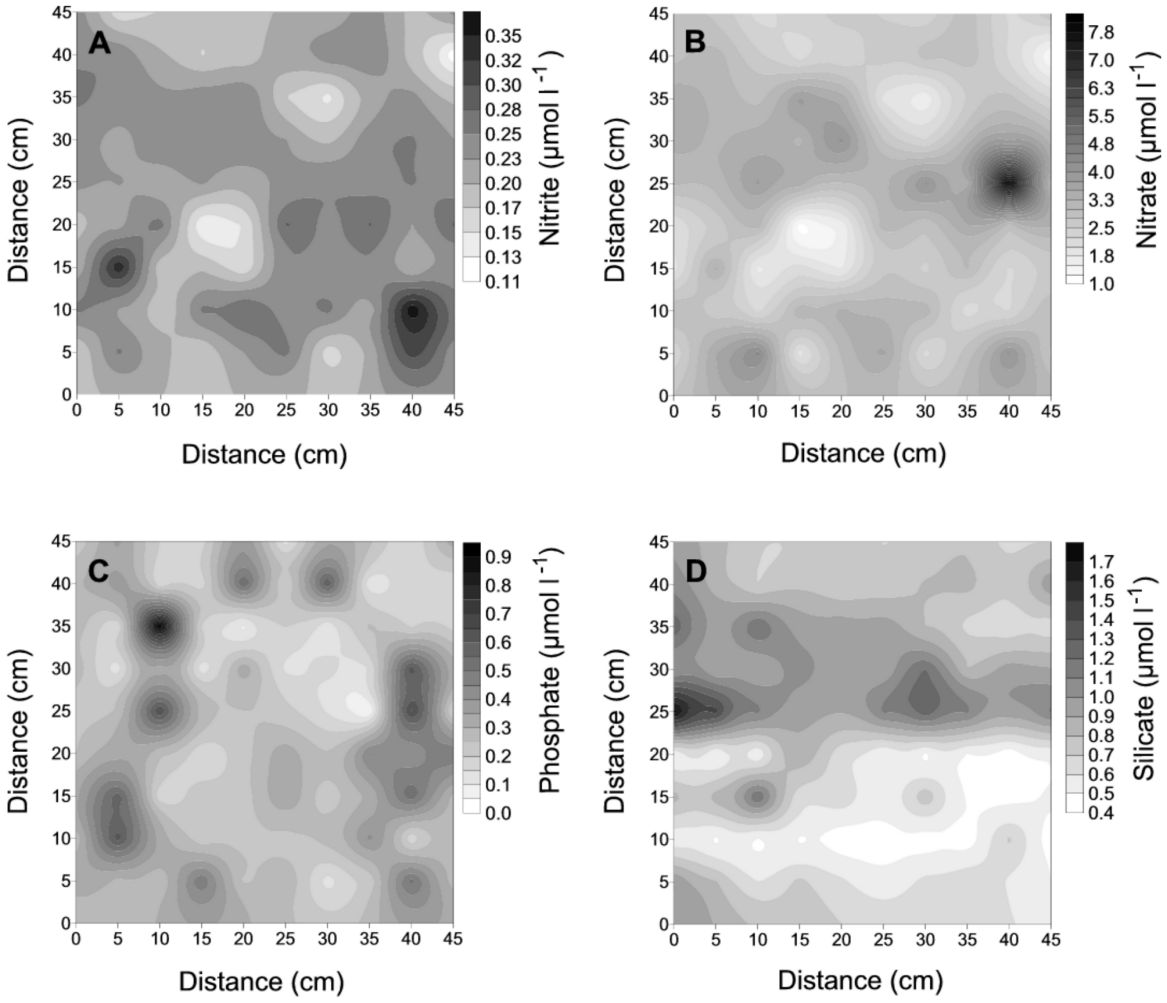
Two-dimensional (x,y) plots of nitrite (a), nitrate (b), phosphate (c) and silicate (d) concentrations estimated from an array of 10×10 syringes of 60 ml simultaneously sampled at a spatial resolution of 5 cm. The strongest changes were 3, 7, 21 and 4-fold, respectively, for nitrite, nitrate, phosphate and silicate, and were observed over distances ranging from 5 cm for silicate (d) to 20 cm for phosphate (c).

To assess the extent of nutrient variation that an individual diatom might experience, we measured inorganic nutrient concentrations in the Eastern English Channel at horizontal intervals ranging from 5 to 45 cm. We found changes over these distances of 2 to 160 times that existed as hotspots on a relatively uniform baseline (Fig. 1). We examined the baseline in two horizontal directions at 5 cm resolution and found centimeter-sized hotspots (Fig. 2) for all nutrients, as well as gradients across the sampling area (Fig. 2c, 2d). These patterns were consistent over the course of the spring bloom, on a seasonal and inter-annual basis. The results show that each nutrient has a distinct and highly variable distribution over distances down to centimeters (Fig. 2).

**Figure 3:**
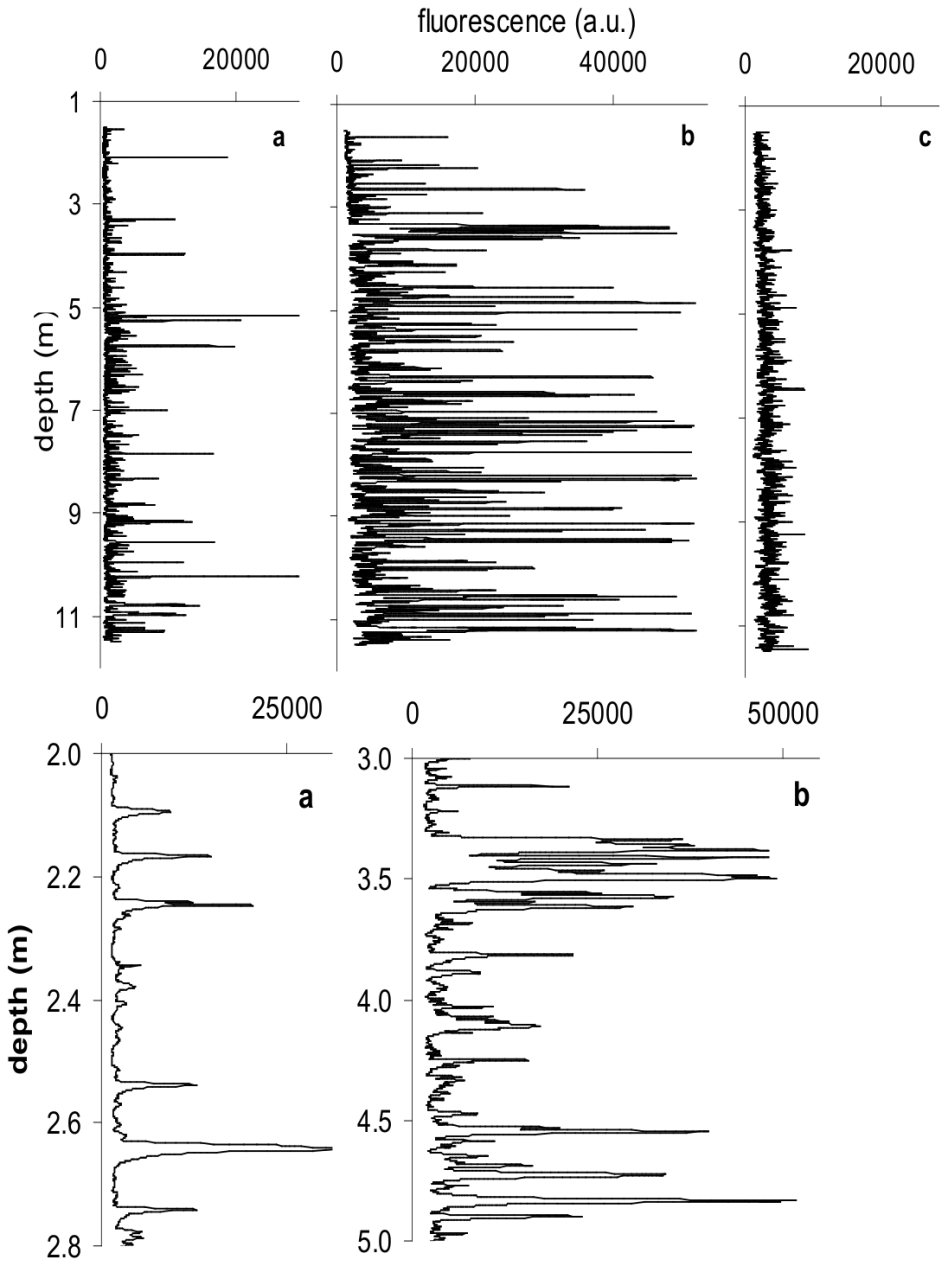
Three representative examples of 90 profiles of chlorophyll fluorescence taken over 3 months in the eastern English Channel. They are from 23/3/04 (a), 26/5/04 (b) and 20/6/04 (c). Fluorescence measurements are in relative arbitrary units (a.u.). Expanded sections of b, showing the regularity of peak spacing (d) and the variety of peak width and height (e). The baseline is 25 times below the highest peak. Noise levels were under 200 a.u.

To further increase resolution and obtain continuous vertical profiles, phytoplankton distributions, as indicated by fluorescence, were used as a proxy for nutrient distributions, based on the reasoning that phytoplankton biomass and nutrient concentration would be inversely related. Fluorescence varied by up to 35 times over 5 cm of depth, and at less than 5 cm, fluorescence variations of 5 to 25 times were common (Fig. 3). More specifically, ninety vertical profiles over 3 months during the spring bloom in the Eastern English Channel showed that the bloom occurred by the proliferation of ubiquitous millimeter to centimeter-scale micropatches of fluorescence, indicating that centimeter scale patches and millimeter scale gradients are common in time as well as space (Fig. 3).

Close examination of micropatches over a season (Fig. 3a–c) showed, closely packed regular spacing and cascades of intensity (Fig. 3d–e). Nutrient and phytoplankton distributions varied down to the measurement resolution, and showed complex internal organization. The fluorescence distribution at the climax of a phytoplankton bloom in the EEC had biomass gradients of 32 times over 5 cm (Fig. 3). At higher resolution, fluorescence intensity changes were 1.5 times per millimeter. The results show extensive heterogeneity in the form of strong nutrient and biomass gradients throughout the water column, and are consistent with the notion that phytoplankton are adapted to the surrounding nutrient and flow environments [16]–[19]. These gradients were used to drive analysis and models of the immediate environment around individual phytoplankton cells.

### Phytoplankton morphologies and uptake

**Figure 4:**
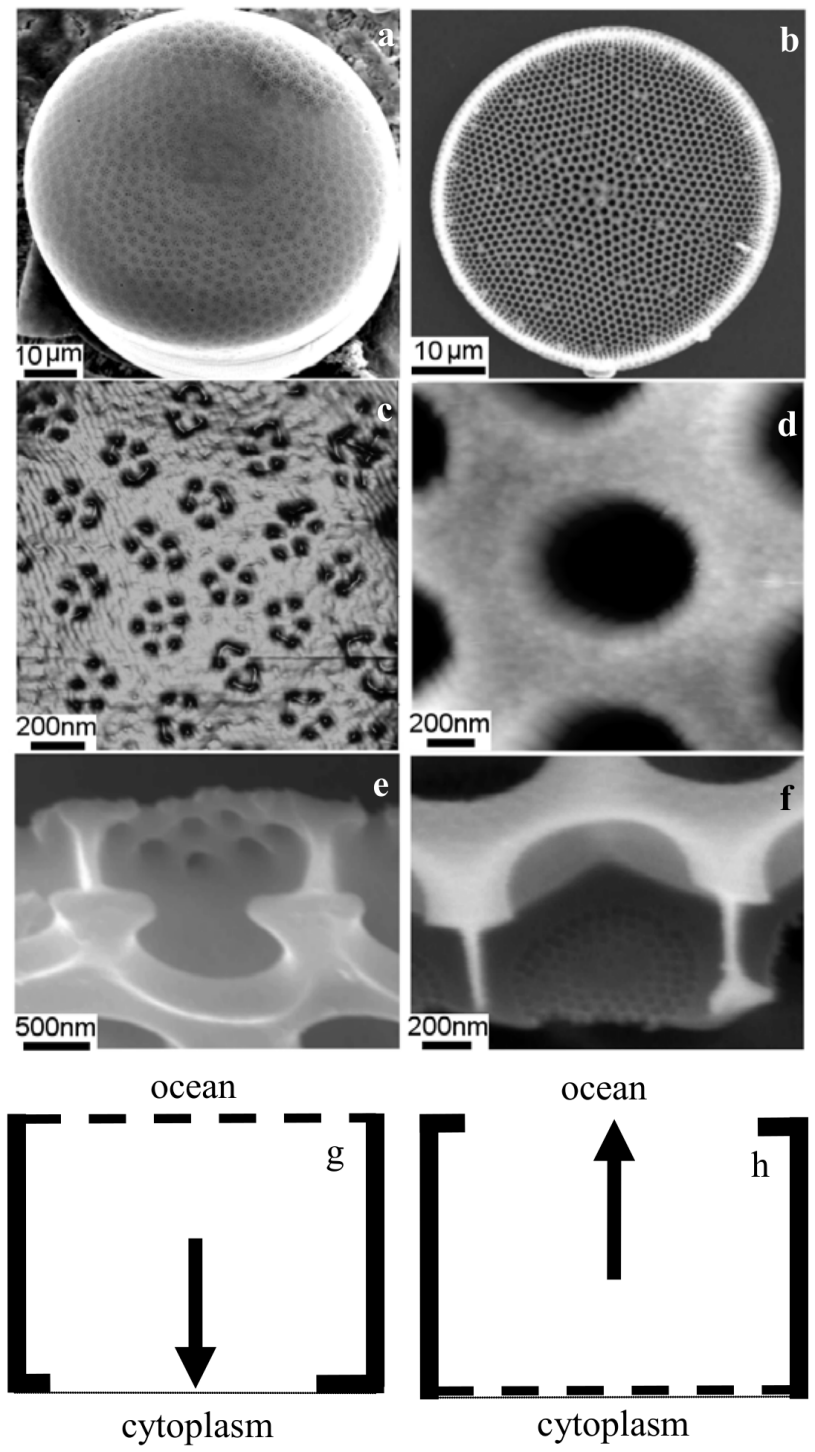
The diatoms *Coscinodiscus* sp. (a–c) and *T. eccentrica* (d–f) with close ups of their outer surfaces (b and e) and cut away sections of the frustule showing trapping (c) and open (f) morphologies. A schematic shows the effects of trapping (g) and open (h) morphologies, where the net nutrient flux (arrows) after a nutrient pulse passes is inwards (g) or outwards (h). The diatoms are 60 and 30 µm across. The large openings are approximately 1 µm and the small openings are 40 nm. The latter are expanded here for graphical clarity. Additional siliceous membranes with intermediate pore sizes may be present in some forms (c).

We looked for adaptations in phytoplankton that we could quantitatively link to the heterogeneous nutrient structure. In particular, we examined the morphology of two commonly occurring diatoms. Our approach was to examine the diffusive resistance of diatom surfaces to nutrients. Electron microscopy and atomic force microscopy imaging showed that the frustules above the cell membranes in *Thalassiosira eccentrica* (Fig. 4a) and *Coscinodiscus* sp. (Fig. 4b) are an array of chambers with different size openings at the top and bottom.

Both diatom species have a hexagonal array of 1 µm wide pores called areolae that penetrate the silica frustules. These pores form chambers above the cell membrane. For *T. eccentrica* there is a silica sieve plate a few tens of nanometers thick with 40 nm wide openings that sit on the cell membrane at the bottom of each chamber. For *Coscinodiscus* sp. the sieve plate sits at the top of the pore away from the cell membrane, creating a chamber with 40 nm holes to the ocean on top and a cell membrane at the bottom.

Qualitatively, the open morphology of *T. eccentrica* (Fig. 4a) provides minimal diffusive resistance to uptake in a homogeneous nutrient environment. However, if the external concentration drops, the net flow of nutrient is away from the membrane because the sieve plate has greater diffusive resistance than the open areolae. In contrast, in the closed morphology of *Coscinodiscus* sp. (Fig. 4b) the outer position of the sieve plate provides greater diffusive resistance than the cell membrane. If the concentration at the sieve plate surface drops, as would be the case in heterogenous nutrient environments, the net diffusion in the chamber remains towards the cell membrane rather than out of the areolar chamber to the ocean.

The observed nutrient gradients were used to drive analytical and numerical nutrient diffusion models (methods). Quantitatively, for the analytical model, *Coscinodiscus* sp. and *T. eccentrica* had diffusion to their respective cell membranes that were 3.8 and 0.5 times the diffusion to the bulk water from the chamber after a nutrient pulse has passed. Using an asymmetrical diffusion equation (methods and ref. 19), the nutrient uptake per unit area for *Coscinodiscus* sp. was 8 times that of *T. eccentrica* after a nutrient pulse passed. In contrast, in a homogeneous nutrient environment *T. eccentrica* uptake was 1.3 times that of *Coscinodiscus* sp. because of a lower overall diffusive resistance.

**Figure 5:**
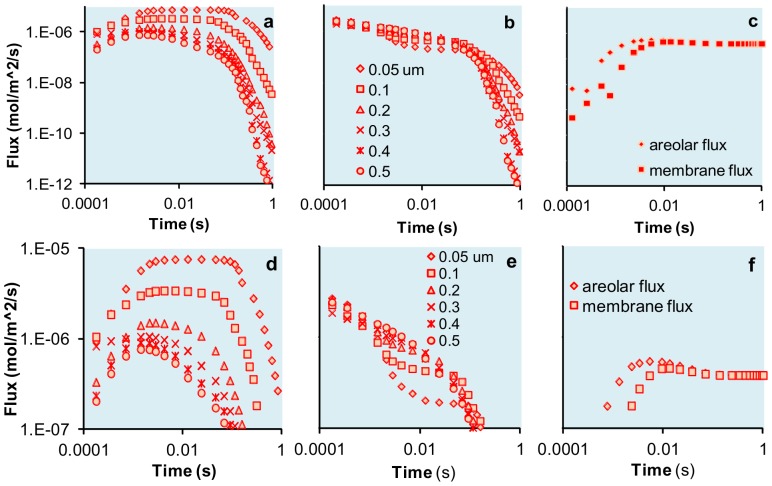
Nutrient fluxes across the aerola and membrane. In (a), the sieve plate is at the top of the aerolar opening as is seen in *Coscindiscus* sp. The different points show equivalent diameters for the open area of the sieve plate. The drop in flux indicates diffusion limitation. In (b), the fluxes are at the membrane surface of *Coscinodiscus* sp. Again, the steep drop approaching 1 s indicates diffusion limitation. In (c) the fluxes are for the open aerola of *T. eccentrica* at the areola and at the membrane. The absence of a drop approaching 1 s in (c) indicates no diffusion limitation. (d) is an expansion of the upper concentrations in (a). The diffusion limitation in (d) and (e) is evident with the drop in fluxes. However, comparison with (f) shows that temporarily the fluxes into the aerolar chamber are higher for the *Coscindiscus* sp. geometry than for the *T. eccentrica* geometry.

A numerical model showed uptake pulses of up to 8×10^−6^ mol/m^2^/s for the *Coscinodiscus* sp. morphology at the sieve plate surface (Fig. 5a, d). The greater area for the membrane surface compared to the sieve plate area resulted in a lower maximum flux of 2×10^−6^ mol/m^2^/s at the membrane, and monotonic decreases over time, rather than distinct peaks (Fig. 5b, e). This was independent of the equivalent sieve plate areas chosen. For all cases, there was a rapid decline as the pulse passed and the chamber emptied. In contrast, the fluxes for the *T. eccentrica* morphology rose over time and reached a stable flux at 5×10^−7^ mol/m^2^/s (Fig. 5c, f).

## Discussion

### Microscale nutrient gradients

Our results demonstrate the existence of nutrient (Figs. 1, 2) and biomass (Fig. 3) gradients that change by orders of magnitude over distances of a few centimeters or less. The data are conservative measures of the range of potential microscale gradients as they are from strongly mixed surface water. Within the context of previous studies [11], [20], [21], these results indicate multiscale nutrient fluctuations down to the separation distance of individual phyto- and bacterioplankters. The significance of these gradients is that plankton are exposed to rapid and steep fluctuations in nutrients in the strongly mixed surface ocean.

Our results show steep gradients down to the limits of measurement resolution. This raises the question of the actual lower limits of fluctuations. Below our smallest observed fluctuations, the distribution of nutrients is a balance between molecular diffusion and advective transport by the smallest turbulent eddies. This advective-diffusive balance tightly constrains our model, and shows how microplankton experience gradients. Specifically, the smallest scale at which advective fluctuations can occur before being eliminated by molecular diffusion is quantitatively given by the Batchelor scale, *B_l_ = *(νD^2^/ε)^0.25^, where ν is kinematic viscosity, D is molecular diffusivity and ε is turbulent energy dissipation [17], [20]. For dissolved ions, *B_l_* is 0.039*l*, where *l* is the length of the smallest turbulent eddies, i.e. the so-called Kolmogorov length scale. In well mixed surface waters the smallest eddies are 1 mm [17], [20], which gives a Batchelor scale of about 40 µm. An average nitrate molecule requires less than 1 s to diffuse that distance. Recent work proposes that the Batchelor scale extends to 10 µm [11], a distance that would require 50 ms to cross by diffusion.

Short distances imply rapid diffusion, which in turn implies rapid nutrient exchange. While the work here derives from theory, there is indirect experimental evidence as well. For example, bulk amino acid turnover times of less than 5 minutes suggest tight nutrient exchange couplings over short distances [22]. In addition to smallest-scale eddy mixing and shear, there is also local nutrient gradient creation. For example, lysis rates due to viral infection of 10% d^−1^ equate to the lysis of 10^5^ cells ml^−1^ d^−1^, meaning that approximately 1 bacterium lyses ml^−1^ s^−1^, adding highly localized nutrient bursts to seawater every second. Bacterial clustering events and viral dynamics ensure that the distribution of lysis events will be highly variable in time and space [23], adding to larger, unevenly distributed, nutrient bursts from zooplankton excretion [24]. This further supports our model assumptions of nutrient pulses of about 1 s. The conclusion is that the surface ocean has heterogeneous nutrient distributions down to the micrometer scale. In contrast to nutrient distributions are typically considered homogeneous below a meter, or even a kilometer [3].

Microplankton behavior, particularly for highly motile bacterioplankton [11], suggests that the nearest neighbor distances will be asymmetric and highly variable. This will be difficult to measure *in situ* because at our peak density the separation distance for homogenously distributed microplankton is 30 µm. This is similar to the Batchelor scale, and is a distance over which swimming, sinking and aggregation can alter a homogeneous distribution in a few seconds. In such a setting, short-term nutrient limitation should play a role in strategies for nutrient competition. The cause of these gradients, and hence the competition will vary in time and space. Chemotactic swarms, shear-dependent clusters, colonies, aggregates and polymer matrix attachment all potentially contribute to gradients in plankton and nutrient distributions [3], [6], [18], [20], [23]. This ‘sea of gradients’ [36] is then the nutrient environment diatoms, and indeed all microplankton, experience.

### Microscale nutrient gradients, surface structure and uptake

Nutrient uptake can lead to depletion out to about 9 cell diameters [16]. This equates to nutrient depletion in approximately 10 s. In the past, local nutrient depletion of any sort has been considered disadvantageous [16]. However, if a cell can constrain diffusion when nutrients are present to increase their own immediate uptake, there may be a competitive advantage in local nutrient depletion. Cells unable to constrain diffusion and transiently store nutrients would experience lower overall uptake as indicated in Fig. 5. The constraining cells may also gain advantage by reducing or eliminating the nutrients available to immediately surrounding competitors.

The mechanistic nutrient uptake explanations proposed here are capable of explaining phenomenological experimental results. In laboratory competition experiments, Peters *et al.* [7] created stirred and unstirred competition experiments, and showed *Thalassiosira* out competed *Coscinodiscus* in a stirred environment; the reverse happened in an unstirred environment. They reported 2 times as many *Coscinodiscus* sp. as *T. pseudonana* in turbulent versus calm conditions, where gradients would be sharp and short-lived. Because *Coscinodiscus* sp. cells are twice as large as *T. pseudonana*, their being twice as many *Coscinodiscus* sp. corresponds to a biomass difference of 8 times [7], which is consistent with the 8-fold greater in uptake reported here for *Coscinodiscus* sp. This similarity is unexpected given the scope in diatoms for variation in vacuole volume, species morphological difference and the obligatory size reduction in the growth process. The commonality of the two studies is the similar frustule structure. That this agreement was arrived at using different methods indicates the potential robustness of frustules structure as a potential physiological control of diatom dynamics and an explanation for the results of Peters *et al*. [7].

### Linking nanoscale surface structures to diatom physiological ecology and species coexistence

Diatoms are highly vacuolated, particularly the genus *Coscinodiscus* [25], with the vacuoles functioning for buoyancy control and nutrient storage [1]. Understanding of the mechanisms of nutrient uptake in a competitive environment is, however, still incomplete. Here, because of our interpretation of the frustule-structure nutrient interaction, we propose that one advantage of the great vacuole volume in *Coscinodiscus* is that it provides storage space that is necessary because its external structure is more efficient at taking up nutrients in certain environments. Motile phytoflagellates, the other large group of marine phytoplankton, rarely have large vacuoles and rigid, fixed external chambers, possibly because the increased cell size caused by vacuoles and external chambers means increased drag. This, in turn, increases the energy expenditure for swimming. Thus, diatom frustules may be linked to their vacuoles through constrained diffusion of nutrients and subsequent storage.

In the highly turbulent environment of the eastern English Channel, the microscale gradients observed in nutrient concentrations are then likely to explain the coexistence of *Coscinodiscus* and *Thalassiosira* as well as other diatom species, through a morphology-based strategy for nutrient competition. Our modeling is simplistic in considering only 2 morphologies and 1 ion, nitrate. The reality of multiple species of diatoms and other microplankton will be considerably more complex. More generally, we propose the testable hypothesis that when diatom species with different surface structure coexist, intense and brief nutrient pulses that are likely to occur under high turbulent conditions [26] would favor diffusion constraining morphologies such as *Coscinodiscus* sp. In contrast, under low turbulent conditions, pulse intensity decreases and duration increases [26], hence favoring diatom species with open morphology such as *T*. *eccentrica*. To demonstrate this as a general principle for diatom frustules, further experimental work and technological developments are needed to assess nutrient fluxes at the smallest temporal and spatial scales.

### Conclusion

In synthesis, it appears that the largest nutrient and biomass changes for phytoplankton occur at the smallest microbial community scales. Our results support the concept of strong strong biomass and nutrient gradients in the ocean. The results here demonstrate one morphologically based strategy for nutrient competition in strong gradients, which is to trap transient pulses near the cell membrane to increase the diffusion to that cell membrane by creating chambers of asymmetric diffusive resistance. The changing slope and frequency of nutrient change probably means that a given morphological adaptation has a short lifetime, giving rise to the species cascade of many coastal marine microbial environments, and helping to explain the morphological and taxonomic diversity in some aquatic microbial systems. This can be quantified by examining the size of the trap that occurs when a given transport process dominates, suggesting particular diffusive regimes have optimal nutrient uptake. Our results indicate that the subtle nanoscale morphological features of marine microbes are tuned to exploit microscale environmental conditions in distinct ways and may play a fundamental role in the competitive interactions that ultimately influence species composition and marine productivity.

## Materials and Methods

Nutrients were measured by standard methods. Fluorescence was taken as a proxy for phytoplankton biomass, and profiles were measured using a high resolution free-fall fluorometer [27] in the EEC. Sampling took place during a protracted spring bloom in April 2004, while the Channel was well mixed from surface to bottom [27] and *Coscinodiscus* sp. and *Thalassiosira* sp. were both members of the phytoplankton community [13].

No specific permits were required for the described field studies. The open ocean area sampled is not privately owned and the study did not involve endangered or protected species.

### Sub-millimeter fluorescence profiles

To begin to approach the Batchelor scale fluctuations that models indicate occur in fully developed turbulence [11] we used a custom-built fluorometer. The fluorometer (FluoroMAP, JFE Advantech Co. Ltd., Kobe) measured pressure, and fluorescence between 640 to 720 nm in a 20 µl sampling volume at 512 Hz [27]. The free-fall speed was 0.25 (±0.02) m s^−1^. Data were taken aboard the *NO Sepia II* (EEC, 50°40′75N-1°31′17E and 50°40′75N-1°24′60E). Hourly CTD profiles were made with a Seabird SBE 25 Sealogger.

Spatial resolution of FluoroMAP at during the sampling period has previously been shown to be 0.54 mm [27]. Comparison of time series of fluorescence from laboratory tests as well as shipboard tests have shown signals for the observed profiles are significantly above the background noise in all cases (Fig. 5 in [27]). Peaks were significantly above troughs and shoulders when the difference was 1,000 arbitrary fluorescence units, which were twice the noise level observed in laboratory calibrations. Data was corrected for change in fall speed using the pressure sensor. Note that the ranges in Figure 3 are either 0 to 25,000 or 0 to 50,000.

### Microscale nutrient distributions

The microscale nutrient concentration was investigated from time series of nitrite and ammonium sampled at 0.5 Hz [14], and from the spatial distribution of nitrate, nitrite, silicate and phosphate taken from 300 samples of 60 ml taken simultaneously at 5 cm intervals using a three-dimensional purpose-designed sampling device [14]. The sampling device consisted of 60 ml syringes in a tubular, stainless steel frame with sampling triggered by a pneumatic system driven by a SCUBA tank. It was tested in the lab, used in the field and has been shown in both cases to minimize disturbance to the water. Orientation and movement relative to the water were controlled to further minimize disturbance and testing was replicated with multiple controls [14].

For the microscale distribution of nitrite and ammonium, water pumped from 0.25 m at 1 m from the hull was directly processed in a Technicon autoanalyzer II. Standard measures minimized mixing artifacts [14]. There was no contamination as indicated by absence of peaks in Fourier space. The linearity of the entire power spectra indicates there was no electronic or processing noise. Temporal scales were converted into spatial scales using the Taylor's theory of Frozen Turbulence using the mean flow velocities observed during the sampling.

The two-dimensional distributions of inorganic nutrients were obtained using a three-dimensional sampling device consisting of three 10×10 arrays of 60 ml syringes, separated at a spatial resolution of 5 cm and mounted on a tubular, stainless steel frame with sampling triggered by a pneumatic system driven by a SCUBA tank [14]. The device was pneumatically operated allowing for the simultaneous collection of 300 samples across three areas of 0.25 m^2^ each. The device was tested in the laboratory [14], used in the field [28], [29] and has been shown in both cases to minimize disturbance to the water. Orientation and movement relative to the water were controlled to further minimize disturbance and testing was replicated with multiple controls as described in Seuront and Menu [14]. Samples were taken from a depth of 1 m at each of the sampling sites, immediately frozen at −20°C after collection, and analysed in an auto-analyser (Alliance Integral Futura). To ensure the ecological relevance of our approach, three dimensional samples were taken monthly from February to June 2004 in the inshore and offshore waters of the English Channel, hence our analysis is based on 10 sets of 300 samples for each.

### Hydrodynamic analysis and turbulent dissipation rates

The turbulent structure of the water column was analyzed to detect stratification that might eliminate isotropy. Every 5 min, current speed and direction were measured at 5, 10 and 15 m with Anderaa current meters. In the absence of any vertical gradient of density, the dynamic stability of the water column was calculated using the total shear, *S*, defined as 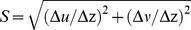, where 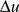 and 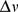 are the vertical gradients in the cross-channel 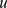 and along-channel 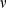 components of the tidal flow over a distance 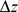.

The dissipation rates of turbulent energy induced by the wind (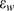,m^2^ s^−3^) and the tidal flow (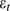, m^2^ s^−3^) were estimated as [14]:
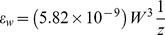
(1)and
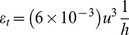
(2)where 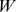 is the wind speed (m s^−1^) and 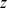 the sampling depth (m), and 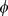, 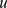 and 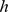 are the fraction of the tidal energy used for vertical mixing, the velocity of the tidal flow (m s^−1^) and the depth of the water column (m), respectively.

### Culturing and AFM

*Coscinodiscus sp.* and *T. eccentrica* were from the CSIRO, Marine Division culture collection (Hobart, Tasmania, Australia). Cultures were grown at 20°C using a 12/12 light/dark cycle. *Coscinodiscus* sp. was cultured in GSE medium and *T. eccentrica* was cultured in Guillard's medium (f/2) for 3 weeks before harvesting and cleaning [30], [31]. Organic material from the frustule surface was removed using concentrated sulphuric acid and surfactant (2% SDS in 100 mM EDTA) to separate the diatom frustules into the valves and girdle bands before storing them in 100% ethanol. Visualization was done by scanning electron microscopy (SEM) and atomic force microscopy (AFM), with SEM images obtained with a Philips XL 30 field-emission scanning electron microscope operated at 2–10 kV after silicon wafers with valves and bands were mounted on microscopy stubs with carbon sticky tape and coated with a thin platinum layer. AFM images were obtained with a Nanoscope IV Multimode SPM (Veeco Corp., Santa Barbara, USA) in air using contact and tapping modes. Oxide-sharpened silicon nitride probes (NP-S, Veeco Corp.) of spring constant *k = *0.15 N/m were used for contact mode experiments. Olympus silicon probes (TESP, Veeco Corp.) were used for tapping mode experiments after precleaning in a water plasma (6 mA source, 0.05 Torr water) for 3 min and characterized using a silicon calibration grating (TGT 01, Silicon-MDT Ltd., Russia). Pores from across the valves were used to determine size, with at least 10 valves of each species imaged, measured and averaged. A minimum of 50 measurements for each size determination were carried out. Measurements were done offline using Nanoscope offline software (Veeco Corp.) and SPIP (Image Metrology, Denmark).

### Analytical spatial trapping of nutrients

Spatial trapping depends on asymmetric diffusion. As a pulse passes a diatom, nutrients equilibrate and then diffuse back to the seawater and to the membrane. The uptake is *I* = *2DsC*, where *D* is molecular diffusivity, *s* is the opening radius and *C* is the background concentration [16]. For comparing the diffusion to the membrane versus to the bulk water we took the ratio of *I_membrane_*/*I_bulk_*, which becomes *I_difference_* = *s_membrane_*/*s_bulk_*. For *Coscinodiscus* sp., the areolae opening radius is 575 nm and the sieve plate opening radius to the bulk water is 22 nm. There are 50 small openings per areolae. Including all 50 openings, the net diffusion to the membrane is 3.8 times greater than the diffusion to the bulk water. For *Thalassiosira eccentrica*, the opening radius to the bulk water is 385 nm, and the membrane opening radii is 21.5 nm, with 72 of the latter per large radius. The result is that the net diffusion to the bulk water is 2.1 times of that to the membrane.

### Numerical spatial trapping of nutrients

The MatLab-derived COMSOL Multiphysics was used to map the distribution of nitrate with the shown geometries using transport of dilute species and creeping flow coupled models. COMSOL has been used to model uptake at a pore and separately to model nutrient uptake by whole diatoms [32], [33]. Here, we combine these two functions and add a chamber immediately below the pore to create an example of constrained diffusion. COMSOL has an established history of use for constrained diffusion problems, but thus far within the cell or for proteins diffusing within a fluid membrane, but not at the cell surface [28], [29]. We followed the general principles of [32] and [33], using [34] to check our use of the finite element method.

Dilute species transport in the non-conserved case was represented by
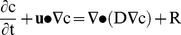
(3)where c is concentration (mol/m^3^), D is the diffusion coefficient (m^2^/s), R is the reaction rate for the species (mol/(m^3^s)) and in this case is used to signify diffusion, **u** is the vector velocity (m/s). Low Reynolds number, incompressible flows are characterized by
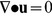
(4)
Equations (3) and (4) were coupled in the MatLab-derived COMSOL Multiphysics 4.0a as a finite element 3 dimensional model. The minimum mesh size was 10^−8^ m. A wall-less channel 10^−6^ m on a side and 3×10^−6^ m long overlaid an aerola 10^−6^ m in diameter. The cylindrical areolar chamber was 0.5×10^−6^ m deep with a flux at the bottom surface of 2×10^−7^ mol/m^2^s. The nutrient was represented as a concentration of 10 µM and a diffusivity of 10^−9^ m^2^/s to mimic nitrate. Pulses were 1 s so as not to exceed the observed nutrient and biomass gradients. Diffusion to the diatom cell membrane was constrained by a second, thin, disk-like cylinder, 10^−8^ m high representing the sieve plate, with diameters indicated in Fig. 5 and placement at either the top of the aerolar chamber for *Coscinodiscus* or at the bottom of the chamber for *T. eccentrica*. Flow was 10^−4^ m/s.

The model was compared to the analytical solution and found to be within 20% of the analytical values for nutrient trapping at the maximum uptake. In addition, the model was checked at 3 grid meshes of 2, 4 and 5×10^−8^ m to eliminate model-driven resonances. Initial benchmarking was done with an absorbative disk in a walless channel to check that the 3D model produced results consistent with model settings, the independent analytical solutions reported here, and that there was confirmation of finite element calculations as per reference [34]. These checks all confirmed that the model represented diffusion in a system representative of nutrients passing over a pore with a recessed chamber below it.

### Conversion by anonymous reviewer

One reviewer found our mks unit of mol/m^2^s unfriendly for some specialists, and to this end provided a conversion. Specifically, consider a flux of 8×10^−6^ mol/m^2^s and a 50 µm diameter cell from our model. Then, calculate the cell nitrogen by assuming a diatom-specific C content and volume of 0.288(cell volume)^0.88^ pg C/cell [35] and a mol∶mol CN ratio of 6.6. This gives 6.4×10^−11^ mol N/cell. Combining the flux with the area of 7.8×10^−9^ m gives a per cell uptake of 6×10^−14^ mol/s, which means 1,000 pulses of 1 s are required to provide enough N for division. *Nota bene*: The calculation is conservative in the sense that the volume is for a sphere. The disk shape for a diatom reduces the volume required for reproduction. Specifically, for a disk 10 µm high the volume is reduced to 33% of a sphere with the same diameter, but the area available for uptake is reduced to only 70% of a sphere, then a more accurate estimate is 500 pulses of 1 s are needed for reproduction. If division is daily this would equate to a pulse every 3 minutes. At a conservative Batchelor scale gradient spacings 200 µm [11], [36] and cells moving relative to the water at 100 µm/s, or about 9 m/d then the encounter rate of a pulse is every 2 s. The range of diatom sinking rates are from 0.1 to >10 m/d [37], [38], a recent, frustule corrected average for disk shaped diatoms is 5 m/d or roughly 60 µm/s [39]. Our value 9 m/d is at the upper range. If we use the lower range then the patch encounters are every 200s, still quite frequent. This suggests rapid reproduction, but ignores competition and depletion.

## Supporting Information

Text S1 Supplementary Information
(DOCX)
