# Supplementary material for: The Role of Diatom Nanostructures in Biasing Diffusion to Improve Uptake in a Patchy Nutrient Environment
Source: PLoS One. 2013 May 7;8(5):e59548. doi: 10.1371/journal.pone.0059548 (PMC3646848; doi:10.1371/journal.pone.0059548)
Supplement: Text S1 — Supplementary Information (DOCX) [file pone.0059548.s001.docx]

Supplementary Information

**Physical description of the immediate diatom environment**

To place this into the more general context of plankton life we further examine their separation distance. The distance between competing phytoplankton cells would be about 1 mm if they were to be randomly spaced (in a Poissonian sense) in three dimensions at the common concentration of 10^3^ cells ml^-1^. This is well above the diffusion-dominated length scale, so there would be physically driven fluctuations at distances shorter than the separation distance. The competing heterotrophic bacterial neighbors would have an even three dimensional spacing of about 100 µm at the common euphotic zone concentration of 10^6^ bacteria ml^-1^. This is similar to the length scale at which diffusion begins to dominate. Considering realistic biological distributions means the cell spacing will vary due to swarming, colony formation, fluid shear, cell division, aggregation, grazing, polymer matrix attachment and lysis [20,21]. These processes will locally increase viscosity and release molecules of varying diffusivities, hence influence the scale of fluctuations. Excretion, grazing, lysis and bacterial remineralisation will regenerate local, short-lived nutrient hotspots that can extend over micrometers to millimeters, to which competing cells can respond [22]. This will further reduce *B_l_*. Thus, diatoms, as well as bacteria, are likely to be exposed to short-lived small scale fluctuations. This also implies rapid nutrient exchange over short distances.

A key factor in competition is constancy or reliability of a resource. For marine microbes, nutrient concentrations vary on multiple time and space scales. For example, in spring, nutrient concentrations are high from winter mixing and light intensities increase. The uptake of nitrate by phytoplankton creates blooms that are major sources of annual marine primary production and intensify nutrient competition by increasing the number of competitors and decreasing the resource [2].

At the scale of individual cells, the extent and impact of nutrient competition are incompletely documented, as are the adaptations to changes in nutrient concentrations. What work that has been done has focused on optimal competitive tuning of high versus low affinity transporters at varying nutrient concentrations [3-5].

To test the convention of uniformity we further modeled the local environment above an individual diatom frustule to understand the relationship between nutrient change and nutrient uptake, with the goal of improving the understanding of the microenvironment where nutrient uptake occurs. We began with modeling because *in situ* uptake measurements through a single diatom frustule pore are well beyond current technology, as are nutrient fluctuation measurements on the second time scale 1 µm above a diatom frustule. To get some nutrient fluctuation estimate with which to drive the model achieve this we began by estimating the size and the extent of nutrient and phytoplankton fluctuations at a the highest currently available spatial resolutions of 5 cm for nutrient distributions, and 0.05 cm for phytoplankton distributions [27]. These are not, which are not *a priori* floors to the fluctuation scale lower limits to the fluctuations, just the current technological limits. However, as mentioned in the discussion of the Batchelor scale, for variation to occur below these scales there must be rapid regeneration of nutrient pulses to compensate for the increasing rapidity of molecular diffusion, otherwise an even, steady state concentration at steady state would results. The evidence for rapid pulses includes excretion and lysis events, which have been observed at scales down to individual microbes [22]. We assumed the magnitude of fluctuations would be similar to those we measured at larger scales based on the reasoning that the greater effectiveness of molecular diffusion at these scales is balanced by the frequent, high concentration nutrient addition events of lysis, grazing and leakage, as well as by the local continuous removal of nutrients by uptake. We point out that for the latter, bacterial surfaces continuously maintain strong gradients through uptake that extend 1-3 µm from their surfaces, and that these gradients, well below the Batchelor scale are probably the smallest steady gradients in the ocean.

For nutrient pulses in the ocean, molecular diffusion balances against transport by the smallest turbulent eddies at the Batchelor scale, which is given as 0.039*l* for dissolved ions, where *l* is the length of the smallest eddies, which in well mixed surface waters range from 1 to 10 mm [10,11,20]. The boundary between transport by diffusion and turbulence is then about 100 µm and corresponds to a time scale of less than 10 s. These are the rough upper boundaries for diffusion dominated transport.

So, strong nutrient gradients occur at scales relevant to the diatom frustule and we take larger scale fluctuations to be representative. Here, there are high frequency nutrient fluctuations over short distances, competition among adjacent neighbours may occur. Here, we show that microscale inorganic nutrient and biomass fluctuations are a common feature of the Eastern English Channel, a coastal environment characterized by high dissipation rates that range from 5×10^-7^ to 5×10^-4^ m^2^ s^-3^ [12]. The high dissipation means that large scale gradients are minimized and that mixing is strong.
